# Hyponatremia in a patient with scleroderma renal crisis: a potential role of activated renin-angiotensin system

**DOI:** 10.1186/1471-2369-13-47

**Published:** 2012-06-27

**Authors:** Hirotaka Fukasawa, Ryuichi Furuya, Sayaka Ishigaki, Naoko Kinoshita, Shinsuke Isobe, Yoshihide Fujigaki

**Affiliations:** 1 Renal Division, Department of Internal Medicine, Iwata City Hospital, 512-3 Ohkubo, Iwata, Shizuoka, 438-8550, Japan; 2 First Department of Medicine, Hamamatsu University School of Medicine, 1-20-1 Handayama, Higashi-ku, Hamamatsu, Shizuoka, 431-3192, Japan

**Keywords:** Hyponatremia, Renin-angiotensin system, Scleroderma renal crisis

## Abstract

**Background:**

Scleroderma renal crisis is an important complication of scleroderma (systemic sclerosis) that is associated with significant morbidity and mortality. On the other hand, hyponatremia has never been reported in patients with scleroderma renal crisis.

**Case presentation:**

A 66-year-old man with scleroderma was admitted to our hospital for an evaluation of renal dysfunction and extreme hypertension. The laboratory evaluation revealed remarkably high plasma renin activity in association with microangiopathic hemolytic anemia, and the anti-RNA polymerase III antibody assessment was positive. The patient was diagnosed with scleroderma renal crisis and was started treatment with enalapril maleate, an angiotensin-converting enzyme inhibitor. During hospitalization, the patient developed symptomatic hyponatremia three times and each laboratory analysis revealed improperly high levels of antidiuretic hormone without signs of extracellular fluid volume depletion as well as remarkably high plasma renin activities and angiotensin levels. However, hyponatremia has not been demonstrated to occur as a result of combined therapy with candesartan cilexetil, an angiotensin II receptor blocker, and aliskiren fumarate, a direct renin inhibitor. The plasma renin activities and angiotensin levels were normalized and the renal function was maintained after treatment.

**Conclusions:**

To our best knowledge, this is the first documented case of scleroderma renal crisis complicated with hyponatremia. This report also suggests that the activated renin-angiotensin system may play a role in the development of hyponatremia and that hyponatremia should be taken into consideration as a rare but possible complication associated with screloderma renal crisis.

## Background

Scleroderma renal crisis (SRC) is an important complication of scleroderma (systemic sclerosis) that is associated with significant morbidity and mortality [1]. Strikingly high levels of plasma renin activity are typically observed accompanying malignant hypertension and it is almost certain that the renin-angiotensin system (RAS) plays a central role in the pathophysiology of SRC. In the late 1970s, angiotensin-converting enzyme (ACE) inhibitors were introduced, and these inhibitors have dramatically improved the outcomes of patients with SRC [2].

Hyponatremia is the most common electrolyte disorder seen in clinical practice [3]. To date, it has been reported that certain ACE inhibitors can cause hyponatremia, although the precise mechanism responsible remains to be elucidated [4].

Here, we report a rare case of SRC complicated with hyponatremia. We also describe the potential relationship between a highly activated RAS and the development of hyponatremia.

## Case presentation

**Figure 1:**
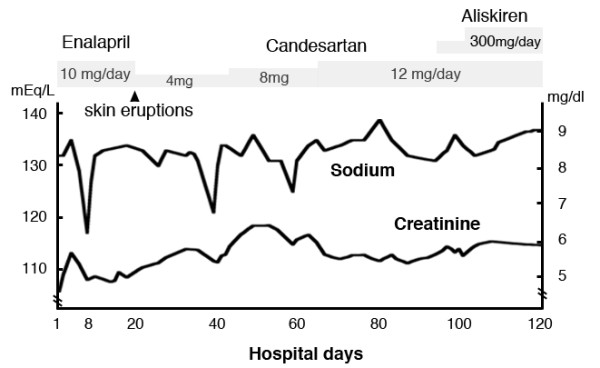
**Clinical course.** The patient was initially treated with enalapril maleate (Enalapril) for SRC. On the 8^th^ day of hospitalization, the patient’s consciousness deteriorated and the serum sodium level was dropped to 116 mEq/L. The level of antidiuretic hormone (ADH) was inappropriately high for the serum osmolarity. The plasma renin activity and angiotensin I level were also extremely high on the same day (see Table 1). After the switch from Enalapril to candesartan cilexetil (Candesartan) due to skin eruptions, hyponatremia similarly developed on 40^th^ and 60^th^ days of hospitalization, respectively. Laboratory data also revealed improperly high levels of ADH, extremely high plasma renin activities and high angiotensin levels. However, hyponatremia had not occurred after the increased dose of Candesartan and the addition of aliskiren fumarate (Aliskiren). The plasma renin activity and angiotensin levels were normalized (see Table 1) and the renal function was preserved. Conversion factors for units: serum creatinine in mg/dl to μmol/L, x 88.4. No conversion is necessary for serum sodium in mEq/L and mmol/L.

A 66-year-old man with a 10-year history of scleroderma was admitted to our hospital for an evaluation of renal dysfunction and accelerated hypertension in May 2010. His renal function and blood pressure had previously been normal. On admission, his blood pressure was 240/128 mmHg and he had hypertensive retinopathy with Keith-Wagner grade III fundoscopic changes, sclerodactyly and superficial ulcers of the fingertips. A laboratory evaluation revealed the following: a blood urea nitrogen (BUN) level of 71 mg/dl (25.3 mmol/L), a serum creatinine level of 4.69 mg/dl (414.6 μmol/L) and a plasma renin activity above 20 ng/ml/hr (above 5.56 ng/L/s, normal range 0.9–2.9 ng/ml/hr) with microangiopathic hemolytic anemia. In addition, the anti-RNA polymerase III antibody test was positive, but the anti-Scl-70 (topoisomerase I) antibody test was negative. From these findings, we diagnosed this patient as having SRC and he was immediately treated with the ACE inhibitor, enalapril maleate (Enalapril) at 10 mg/day. After starting the treatment, his blood pressure decreased and renal dysfunction partially improved (Figure 1).

On the 8^th^ day of hospitalization, his consciousness suddenly deteriorated and urgent laboratory findings revealed that his serum sodium level had decreased from 131 mEq/L (131 mmol/L) at the time of admission to 116 mEq/L (116 mmol/L), his urine osmolarity (332 mOsm/kg = mmol/kg) was greater than his serum osmolarity (261 mOsm/kg) and the level of antidiuretic hormone (ADH) was 6.1 pg/ml (5.6 pmol/L), which was improperly high for the indicated serum osmolarity. His thyroid function was normal and adrenal insufficiency was ruled out. There were no signs of extracellular fluid volume depletion and the results of a brain computed tomography (CT)-scan were normal. Although these data indicated that his hyponatremia was caused via the improper secretion of ADH, the patient did not fulfill the criteria for a diagnosis of the syndrome of inappropriate secretion of ADH (SIADH) due to the co-existence of renal dysfunction. It was also found that the plasma renin activity and angiotensin I levels were extremely high on the 8^th^ day of hospitalization (Table 1). His hyponatremia was treated with water restriction and with the infusion of hypertonic saline.

**Table 1:** Levels of serum sodium, RAS components and ADH at the time of and after admission

	**On admission**	**Day 8 (1st)**	**Day 40 (2nd)**	**Day 60 (3rd)**	**Day 110**
Medication		Enalapril	Candesartan 4 mg	Candesartan 8 mg	Candesartan 12 mg + Aliskiren
Sodium (mEq/L)	131	116	120	124	136
Renin (0.3-2.9 ng/ml/hr)	> 20	> 20	> 20	> 20	2.2
Angiotensin I (< 110 pg/ml)	210	1100	350	560	< 30
Angiotensin II (< 22 pg/ml)	330	41	1500	1100	27
Aldosterone (2.9-15.9 ng/dl)	18.5	14.0	14.0	7.87	3.87
ADH (pg/ml)	Not determined	6.1	0.9	1.0	Not determined
Serum creatinine (mg/dl)	4.69	5.03	5.58	6.06	6.04
Body weight (kg)	Not determined	49.1	46.9	48.1	46.1

Definition of the abbreviations: RAS, renin-angiotensin system, ADH, antidiuretic hormone, Renin, plasma renin activity. Numerical values in each parenthesis indicate normal values.

*Note:* Conversion factors for units: plasma renin activity in ng/ml/hr to ng/L/s, x 0.2778; angiotensin I in pg/ml to fmol/ml, x 0.772; angiotensin II in pg/ml to fmol/ml, x 0.956; aldosterone in ng/dl to nmol/L, x 0.02774; ADH in pg/ml to pmol/L, x 0.923; serum creatinine in mg/dl to mmol/L, x 88.4. No conversion is necessary for serum sodium in mEq/L and mmol/L.

On the 19^th^ day of hospitalization, he exhibited skin eruptions that were an adverse effect of the Enalapril treatment. Then, Enalapril was replaced with candesartan cilexetil (Candesartan) at a dose of 4 mg/day. After this replacement with Candesartan, he developed hyponatremia twice with similar symptoms on the 40^th^ and 60^th^ day of hospitalization, respectively (Figure 1). Laboratory data also revealed improperly high levels of ADH, extremely high plasma renin activity and high angiotensin levels on the 40^th^ and 60^th^ day of hospitalization (Table 1). However, after the dose of Candesartan was increased to 12 mg/day and aliskiren fumarate (Aliskiren) at 300 mg/day was also included in the treatment regimen, his serum sodium levels were normalized and were maintained within the normal range thereafter. His plasma renin activity and angiotensin levels were also normalized and his renal function was preserved.

## Conclusions

Here, we report a rare case of SRC complicated with hyponatremia. To our best knowledge, this is the first case to be described in the literature. Each episode of hyponatremia was accompanied with improperly high levels of ADH, extremely high plasma renin activities and high angiotensin levels without signs of extracellular fluid volume depletion. Furthermore, hyponatremia was prevented via the strong blockade of RAS using a therapeutic combination of the angiotensin II receptor blocker, Candesartan, and the direct renin inhibitor, Aliskiren. Then, it was suggested that the activated RAS might have played an important role in the development of this case of hyponatremia.

To date, there is clear evidence supporting the existence of the central RAS, which is independent of the peripheral RAS [5,6]. Moreover, mechanisms and components required for the formation of angiotensin II have been identified in the brain [6,7]. In accordance with these findings, it has been reported that peripheral angiotensins were unable to penetrate the blood–brain barrier (BBB) under normal physiological conditions [8]. It has also been reported that Enalapril [9], Candesartan [8] and Aliskiren (unpublished data by Novartis Pharmaceuticals Corporation) were unable to penetrate the BBB.

**Figure 2:**
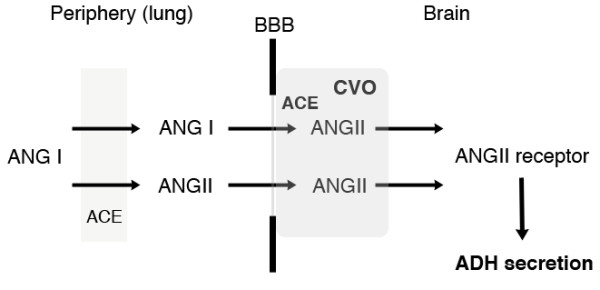
**Diagram of the potential relationship between peripheral and central renin-angiotensin systems and ADH secretion.** Angiotensin II (ANGII) in the peripheral circulation is accessible to circumventricular organs (CVOs), which lack the blood–brain barrier (BBB). Within CVOs, high levels of ANGII (for example, via the infusion of ANGII) can stimulate the ADH secretion via ANGII receptors in the brain. Most of the blood-borne angiotensin I (ANG I) is converted into ANGII at the lungs by the angiotensin-converting enzyme (ACE) under physiological conditions, although the rate of this conversion process is decreased under pathological conditions. Because the activity of ACE in CVOs is much higher than that in the lungs, the redundant ANG I may be converted to ANGII locally and this converted (and high levels of) ANGII may then stimulate the ADH secretion.

On the other hand, it has been reported that high levels of angiotensin II in the peripheral circulation (for example, via the infusion of angiotensin II) could affect the central actions including the secretion of ADH [10]. The effects of high levels of angiotensin II on the hypothalamic-neurohypophysial system are mediated via circumventricular organs (CVOs), such as the subfornical organ and the organum vasculosum of the lamina terminalis. These organs anatomically lack the BBB, and as a result, angiotensins in the peripheral circulation are accessible to these sites [11]. Accordingly, it is likely that supra-physiological levels of angiotensin II were involved in the improper secretion of ADH resulting from the anatomical characteristics of CVOs and these factors subsequently led the development of hyponatremia in our case (Figure 2).

Circulating angiotensin I is converted into angiotensin II by ACE mainly in the lungs, and ACE inhibitors can block this conversion process. In fact, the level of angiotensin I, but not angiotensin II, was remarkably high at the time when the first episode of hyponatremia developed on the 8^th^ day of hospitalization, because the patient was treated with Enalapril, an ACE inhibitor (Table 1). Interestingly, much higher ACE activities have been identified in CVOs compared to those in lungs [12] and Thunhorst *et al.*[13] showed that the peripheral ACE blockade with anti-hypertensive doses could not inhibit the ACE activities in CVOs. Therefore, it was possible that the remarkably high levels of angiotensin I reached in CVOs were locally converted into angiotensin II, that the converted (and likely high levels of) angiotensin II stimulated ADH secretion, and that the first episode of hyponatremia finally developed on the 8^th^ day of hospitalization.

However, we cannot exclude the following alternative possibilities: (i) that the disturbance of free water excretion due to renal dysfunction co-existed also played a role in the development of hyponatremia and (ii) that the permeability of the BBB for angiotensins was altered due to the pathological condition of SRC.

In conclusion, we experienced this rare case of SRC complicated with hyponatremia. Further studies are required to clarify the precise mechanism responsible for the association between SRC and hyponatremia, although the activated RAS may play an important role in the development of hyponatremia in this case.

## Consent

Written informed consent was obtained from the patient for publication of this Case report and any accompanying images. A copy of the written consent is available for review by the Series Editor of this journal.

## Abbreviations

ACE, Angiotensin converting enzyme; ADH, Antidiuretic hormone; BBB, Blood–brain barrier; BUN, Blood urea nitrogen; CT, Computed tomography; CVO, Circumventricular organ; RAS, Renin-angiotensin system; SIADH, Syndrome of inappropriate of ADH secretion; SRC, Scleroderma renal crisis.

## Competing interests

'The authors declare that they have no competing interests.

## Authors’ contributions

HF, SaI, NK and ShI treated the patient. HF wrote the first draft and also evaluated the data. RF and YF wrote the final draft. All authors reviewed the final version of this manuscript.

## Acknowledgements

None

## Pre-publication history

The pre-publication history for this paper can be accessed here:

http://www.biomedcentral.com/1471-2369/13/47/prepub
